# Discovery of the Potential Biomarkers for Discrimination between *Hedyotis diffusa* and *Hedyotis corymbosa* by UPLC-QTOF/MS Metabolome Analysis

**DOI:** 10.3390/molecules23071525

**Published:** 2018-06-25

**Authors:** Yaru Wang, Cuizhu Wang, Hongqiang Lin, Yunhe Liu, Yameng Li, Yan Zhao, Pingya Li, Jinping Liu

**Affiliations:** 1 School of Pharmaceutical Sciences, Jilin University, Fujin Road 1266, Changchun 130021, China; ywhxwyr123@163.com (Y.W.); wangcz15@mails.jlu.edu.cn (C.W.); linhq17@mails.jlu.edu.cn (H.L.); lyh133700@163.com (Yu.L.); liyameng2018@163.com (Ya.L.); lipy@jlu.edu.cn (P.L.); 2 College of Chinese Medicinal Materials, Jilin Agriculture University, Xincheng Street 2888, Changchun 130118, China; zhaoyan@jlau.edu.cn

**Keywords:** *Hedyotis diffuse* Willd., *Hedyotis corymbosa* (L.) Lam., chemical components, metabolomic analysis

## Abstract

*Hedyotis diffuse* Willd. (HD) and *Hedyotis corymbosa* (L.) Lam. (HC), two closely related species of the same genus, are both used for health benefits and disease prevention in China. HC is also indiscriminately sold as HD in the wholesale chain and food markets. This confusion has led to a growing concern about their identification and quality evaluation. In order to further understand the molecular diversification between them, we focus on the screening of chemical components and the analysis of non-targeted metabolites. In this study, UPLC-QTOF-MS^E^, UNIFI platform and multivariate statistical analyses were used to profile them. Firstly, a total of 113 compounds, including 80 shared chemical constituents of the two plants, were identified from HC and HD by using the UNIFI platform. Secondly, the differences between two herbs were highlighted with the comparative analysis. As a result, a total of 33 robust biomarkers enabling the differentiation were discovered by using multivariate statistical analyses. For HC, there were 18 potential biomarkers (either the contents were much greater than in HD or being detected only in HC) including three iridoids, eight flavonoids, two tannins, two ketones, one alcohol and two monoterpenes. For HD, there were15 potential biomarkers (either the contents were much greater than in HC or being detected only in HD) including two iridoids, eight flavonoids, one tannin, one ketone, and three anthraquinones. With a comprehensive consideration of the contents or the MS responses of the chemical composition, Hedycoryside A and B, detected only in HC, could be used for rapid identification of HC. The compounds 1,3-dihydroxy-2-methylanthraquinone and 2-hydroxy-3-methylanthraquinone, detected only in HD, could be used for rapid identification of that plant. The systematic comparison of similarities and differences between two confusing Chinese herbs will provide reliable characterization profiles to clarify the pharmacological fundamental substances. HC should not be used as the substitute of HD.

## 1. Introduction

*Hedyotis diffuse* Willd. (HD) is a well-known Chinese folk-medicine with a spectrum of pharmacological activities, including anti-cancer, antioxidant, anti-inflammatory, anti-fibroblast, immunomodulatory and neuroprotective effects, especially the anti-cancer effect in practice [1]. Almost 200 compounds have been identified in HD, including iridoids, flavonoids, anthraquinones, phenylpropanoids, phenolics and their derivatives, sphingolipids, volatile oils and miscellaneous compounds [1,2,3].

*Hedyotis corymbosa* (L.) Lam. (HC), another species of the same genus, is also used interchangeably in China as a health supplement and for disease prevention. It is reported to possess antioxidant [4,5], anti-inflammatory [6], hepatoprotective [7,8], antitumor [9,10], antimalarial [11] and anti-nociceptive [12] activities. Iridoids, carboxylic acids, flavonoids, phenolics and their derivatives, triterpenes, anthranquinones and coumarins were isolated from HC [13,14,15]. Iridoid glycosides were reported as the main constituents [16]. Oleanolic acid and ursolic acid were also considered as biologically active ingredients [17,18].

HD and HC are closely related species of the Rubiaceae family. Due to their similar morphology, they are often mixed up. Recently, a systematic survey on confusable Chinese herbal medicines has revealed that HC is indiscriminately sold as HD in wholesale markets or food markets [19]. This confusion in the market has led to a growing concern about the identification and quality evaluation of HD and HC.

Several methods using various techniques have been established to distinguish between these two species, such as loop-mediated isothermal amplification technique (LAMP) [20], fluorescence microscopy [21], thin layer chromatography (TLC) [22], DNA sequencing of the complete internal transcribed spacer region and chemical analysis [23], phylogenetic utility of nuclear ribosomal DNA (nrDNA) internal transcribed spacers (ITS) [24], high-performance liquid chromatography (HPLC) [25], etc. As a result, markers such as hedyotiscone A [22], scandoside methyl ester [25], (9*R*,10*S*,7*E*)-6,9,10-trihydroxyoctadec-7-enoic acid [26] for HC, 6-*O*-(*E*)-*p*-coumaroyl scandoside methyl ester [23,25], (10*S*)-hydroxypheophytin a [23], 6-*O*-(*E*)-*p*-coumaroyl scandoside methyl ester-10-methyl ether and 6-*O*-*p*-feruloyl scandoside methyl ester [25] for HD have been found. The UPLC-UV (detection wavelength at 254 nm) fingerprint of HC was also established to distinguish it from HD [27]. The contents of oleanolic acid and ursolic acid were significantly different [28].

Untargeted metabolomics, with the ability to profile diverse classes of metabolites, is primarily used to compare the overall small-molecule metabolites of different samples [29]. It is mainly applied in metabolites identification through mass-based search strategy followed by manual or automated verification. The combination of ultra-high performance liquid chromatography (UPLC) separation, quadrupole time-of-flight tandem mass spectrometry (Q/TOF-MS) detection and the automated data processing software UNIFI with a scientific library is frequently applied in the characterization of chemical constituents of herbal medicines [30,31,32,33] and traditional Chinese medicine injection recently [34]. High-resolution tandem mass spectrum can provide an accurate and specific mass when the coeluting components possess different *m*/*z* values. UNIFI, a high throughput, comprehensive, simple and efficient platform, offers the approach to integrate data acquisition, data mining, library searching and report generation. The Traditional Medicine Library within the platform contains more than 6000 compounds from 600 herbs.

The aim of the study was search for potential biomarkers in order to systematically screen chemical components and the non-targeted metabolomic analysis of the two species, and in turn providing the basis for establishment of HC and HD quality criterion in the future. UPLC-QTOF-MS^E^, UNIFI platform and multivariate statistical analyses, such as principal component analysis (PCA) and orthogonal partial least squares discriminant analysis (OPLS-DA) were used to profile these two herbs. The established method could enable us to find the similarities and differences between them, and provide data for the establishment of HC and HD quality criterion in the future. This comprehensive and unique phytochemical profile study revealed the structural diversity of secondary metabolites and the different patterns in HC and HD. The method developed in this study can be used as a standard protocol for identifying and discriminating species of HC and HD.

## 2. Experimental

### 2.1. Materials and Reagents

HC and HD were purchased from herbal markets or collected from their respective cultivation areas in China (Table 1). The corresponding voucher specimens had been deposited in the Research Center of Natural Drug, School of Pharmaceutical Sciences, Jilin University, China. All the HC and HD samples were identified with the macroscopic and microscopic characters according to the *Standard of Chinese Medicinal Materials in Guangdong Province* (2004 Edition) and the *Standard of Chinese Medicinal Materials in Shaanxi Province* (2015 Edition). In these Standards, the identified methods only focus on the different macroscopic and microscopic characters. As the chemical constitutes are concerned, both oleanolic acid and ursolic acid are used to quality control. That is to say, there are no biomarkers to distinguish HC from HD.

Acetonitrile and methanol were UPLC-MS pure grade (Fisher Chemical Company, Geel, Belgium). Formic acid for UPLC was purchased from Sigma-Aldrich Company (St. Louis, MO, USA). Deionized water was purified using a Millipore water purification system (Millipore, Billerica, MA, USA). All other chemicals were of analytical grade. For reference substance, ursolic acid (110742-201622), citric acid (111679-201602), chlorogenic-acid (110753-201716), geniposide (110749-201718), luteolin 7-*O*-*β*-d-glucopyranoside (111968-201602), rutin (100080-201409), quercetin (100081-201610), kaempferol (110861-201611), hesperidin (110721-201617) were purchased from the National Institutes for Food and Drug Control (Beijing, China). Scandoside (20170503), alizarin 1-methyl ether (20170608) were purchased from Nanjing DASF Biotechnology Co., Ltd. (Nanjing, China). Scandoside methyl ester (20171001), 5,6,7,4′-tetramethoxyflavone (20171011), geniposidic acid (20171024) were purchased from Sichuan Weikeqi Biotechnology Co., Ltd. (Chengdu, China). 6-Methoxy-8-methylcoumarin (16018), sanlengdiphenyllactone (15025) were provided by the Research Center of Natural Drugs, School of Pharmaceutical Sciences, Jilin University, China.

### 2.2. Sample Preparation and Extraction

All the whole plants, including HC (HC1~HC10) and HD (HD1~HD10), were air-dried, grinded and sieved (40 mesh) to get the homogeneous powder respectively. Then, the powder of 20 samples (200 mg per sample) were extracted respectively with 80% methanol (2L × 3) at 80 °C for three times (3 h each time) with the reflux method. The extraction procedure is repeated until the extracted solution is colorless. After filteration, the extracts of each sample were combined, concentrated and evaporate to dryness. As a result, 20 desiccated extract powders were obtained. Each powder was dissolved in 1.0 mL of 80% methanol. Subsequently, each methanolic solution was filtered and injected directly into the UPLC system. The volume injected of each sample was 2 μL for each run. Furthermore, the methanol blank were run with the same gradient program between two samples during the whole sample list. The wash volume between injections was enough for avoiding carry over. Meanwhile, 20-μL aliquots of each HD and HC sample were mixed to obtain a quality control (QC) sample, which contained all of the components in the analysis. The QC sample was run every five samples to monitor the stability of the system.

### 2.3. Ultra-High Performance Liquid Chromatography with Quadrupole Time-of-Flight Tandem Mass Spectrometry (UPLC-QTOF-MS)

The separation and MS detection of components were performed on a Waters Xevo G2-XS QTOF mass spectrometer (Waters Co., Milford, MA, USA) connected to the UPLC system through an electrospray ionization (ESI) interface. UV wavelength did not trigger the MS detection of components. The column used was an ACQUITY UPLC BEH C_18_ (100 mm × 2.1 mm, 1.7 μm) from Waters Corporation (Milford, MA, USA). The mobile phases consisted of eluent A (0.1% formic acid in water, *v*/*v*) and eluent B (0.1% formic acid in acetonitrile, *v*/*v*) with a flow rate of 0.4 mL/min following a liner gradient program: 10% B from 0 to 2 min, 10–90% B from 2 to 25 min, 90% B from 25 to 26 min and 90–10% B from 26 to 26.1 min. The temperature of the UPLC column and sample was set at 30 °C and 15 °C. Mixtures of 10/90 and 90/10 water/acetonitrile were used as the strong wash and the weak wash solvent respectively. The optimized instrumental parameters were as follows: capillary voltage floating at 2.6 kV (ESI^+^) or 2.2 kV (ESI^−^), cone voltage at 40 V, source temperature at 150 °C, desolvation temperature at 400 °C, cone gas flow at 50 L/h and desolvation gas flow at 800 L/h. In MS^E^ mode, collision energy of low energy function was set to 6 V, while ramp collision energy of high energy function was set to 20–40 V. Each sample was analyzed by UPLC-QTOF-MS^E^ mode; data acquisition was performed via the mass spectrometer by rapidly switching from a low-collision energy (CE) scan to a high-CE scan during a single LC run. The low-CE experiment provides information about the intact molecular ion, e.g., [M+H]^+^, while the high-CE scan generates fragment ion information. Alignment of the low-CE and high-CE data is automatically performed by the software. To ensure mass accuracy and reproducibility, the mass spectrometer was calibrated over a range of 100–1200 Da with sodium formate. Leucine enkephalin was used as external reference of Lock Spray™ infused at a constant flow of 10 μL/min. In addition, MassLynx data were recorded in continuous mode during acquisition.

### 2.4. Chemical Information Database for the Components of HC and HD

In addition to the Waters Traditional Medicine Library in the UNIFI software, a systematic investigation of chemical constituents was conducted. A self-built database of compounds isolated from HC and HD was established by searching online databases such as China Journals of Full-Text Database (CNKI), PubMed, Medline, Web of Science and ChemSpider. The name, molecular formula and structure of components from HC and HD were obtained in the database.

### 2.5. Data Analysis by UNIFI Platform

Data analysis was performed on UNIFI 1.7.0 software (Waters, Manchester, UK). Emphasis was put on analyzing structural characteristics and MS fragmentation behaviors, especially for characteristic fragments. Minimum peak area of 200 was set for 2D peak detection. The peak intensity of high energy over 200 counts and the peak intensity of low energy over 1000 counts were the selected parameters in 3D peak detection. A margin of error up to 5 ppm for identified compounds was allowed. We selected positive adducts containing +H and +Na and negative adducts including +COOH and −H. For exact mass accuracy, with leucine enkaplin as the reference compound, [M+H]^+^ 556.2766 was used for positive ion and [M−H]^−^ 554.2620 was used for negative ion in the UNIFI platform.

The MS raw data were processed using the streamlined workflow of UNIFI software to quickly identify the chemical components that met the match criteria with the Traditional Medicine Library. Firstly, an in-house scientific library was created including the information of chemical components from the target herbs based on the literature, saved as Mol file format, and then, the newly built library was imported into the analysis method, in virtue of some compounds being missing in the Traditional Medicine Library. Secondly, the raw data was compressed by Waters Compression and Archival Tool v1.10 and imported into the software. Thirdly, automated screening and identification were performed by the UNIFI platform instead of manually extracting each individual chromatographic peak, calculating the elementary composition and then analyzing MS fragmentation behaviors. Fourthly, we set up a filter to refine results, being mass error between −5 and 5 ppm, and additionally, response value greater than 6000. Finally, further verification of compounds by comparison with retention time of reference substances and characteristic MS fragmentation patterns reported in literature was carried out. After processing and filtering of the data by UNIFI, all selected components were listed for further verification, including information such as compound name, chemical structure, mass error, adducts, response, extracting ion chromatograms and spectra of low energy and high energy. The components were listed by descending response order and confirmed by reference substances or comparison with literatures. 

### 2.6. Metabonomics Analysis

MarkerLynx XS V4.1 software (Waters, Manchester, UK) was used to process the raw data for alignment, deconvolution, data reduction, etc. As a result, the list of mass and retention time pairs with corresponding intensities for all the detected peaks from each data file. The main parameters were as follows: retention time range 0–26 min, mass range 100–1200 Da, mass tolerance 0.10, minimum intensity 5%, marker intensity threshold 2000 counts, mass window 0.10, retention time window 0.20, and noise elimination level 6. Furthermore, also with the MarkerLynx XS V4.1 software, principle component analysis (PCA) and orthogonal projections to latent structures discriminant analysis (OPLS-DA) were applied to analyze the above resulting data. Whether these two species are different would depend on the separation between HD and HC groups. The obvious separation in PCA score plots means they are differentiated. The supervised pattern recognition approach OPLS-DA can visualize and depict general metabolic variation between two groups. To identify the metabolites contributing to the discrimination, S-plots and VIP-plots were obtained via OPLS-DA analysis to find potential biomarkers that significantly contributed to the difference among HC and HD. Each spot in S-plots represents a variance. The importance of each variance to classification is determined by the value of variable importance in the projection (VIP) and metabolites with VIP value above 2.0 were considered as potential markers.

## 3. Results

### 3.1. Identification of Components from HC and HD

**Figure 1:**
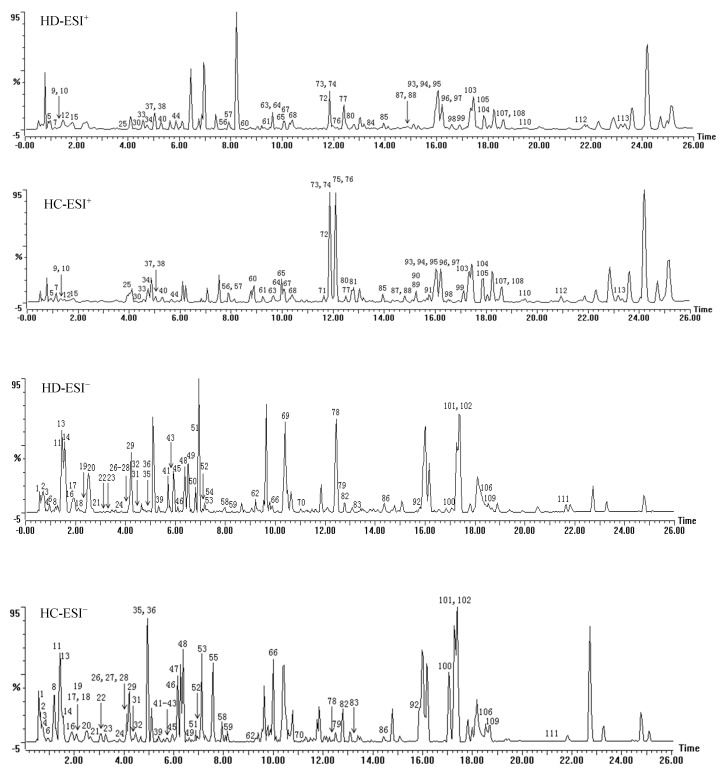
The representative base peak intensity (BPI) chromatograms of HD and HC in positive mode (ESI^+^) and negative mode (ESI^−^). (The character “,” represent the meaning of “and”).

**Figure 2:**
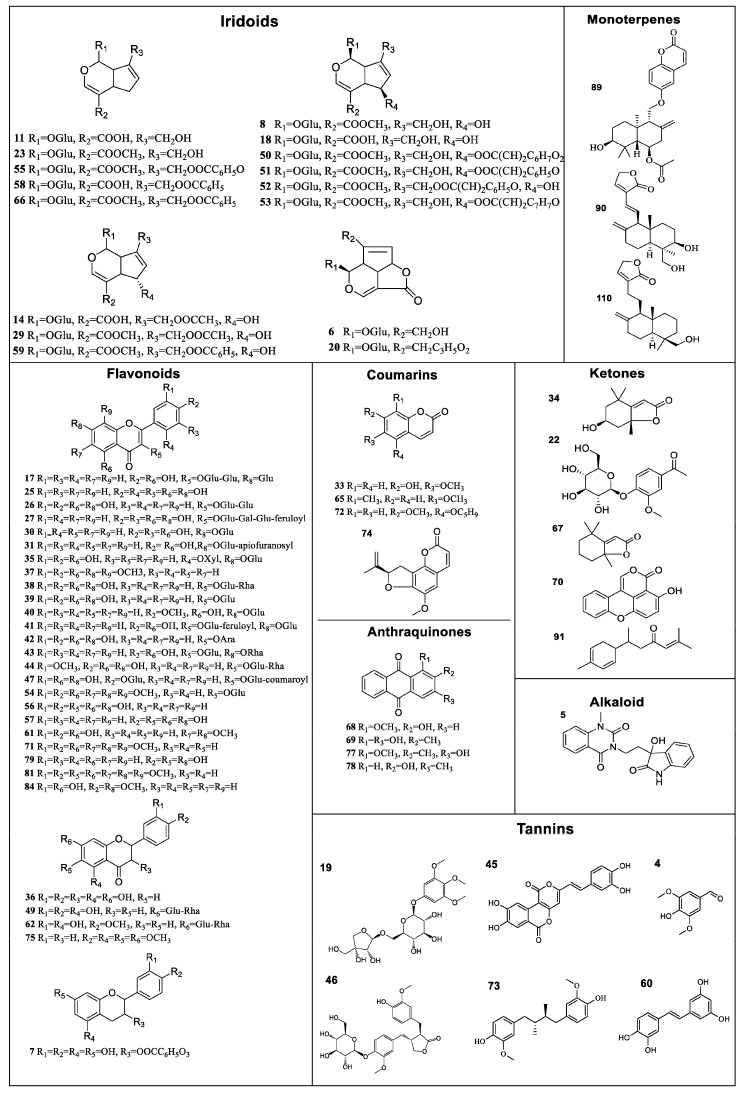

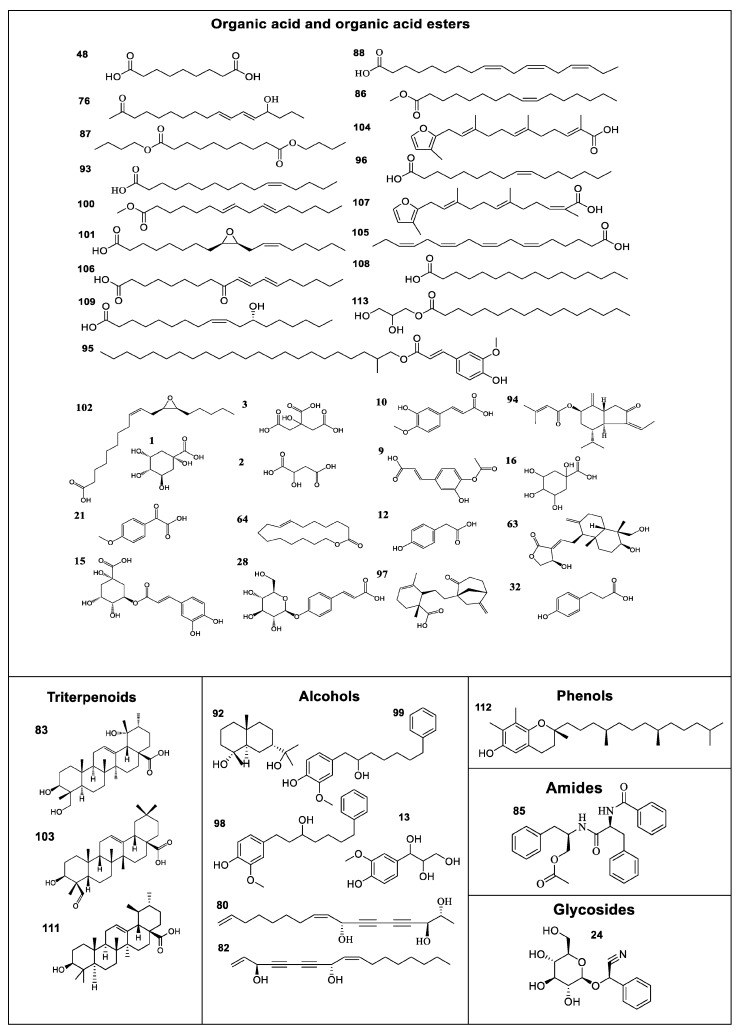
Chemical structures of compounds identified in HD and HC.

A total of 113 compounds were identified or tentatively characterized in both positive and negative mode from HC and HD (Table 2), the base peak intensity (BPI) chromatograms are shown in Figure 1, and their chemical structures are shown in Figure 2. In HC and HD 109 and 104 compounds were characterized, respectively. Both herbs are rich in natural components with various structural patterns, including iridoids, flavonoids, organic acids and organic acid esters, tannins, alcohols, ketones, coumarins, anthraquinones, monoterpenes, triterpenoids, etc. Some of these compounds have isomers may be distinguished based on characteristic MS fragmentation patterns reported in literature, or comparison of retention times to reference substances.

80 common constituents were identified from HC and HD. Among them, there were eleven iridoids (compounds **6**, **8**, **11**, **14**, **18**, **20**, **29**, **51**, **53**, **58** and **59**), thirteen flavonoids (compounds **7**, **17**, **25**, **26**, **27**, **31**, **36**, **37**, **38**, **39**, **43**, **56** and **61**), one monoterpene (compound **10**), one anthraquinone (compound **68**), two ketones (compounds **34** and **67**), three tannins (compounds **4**, **73** and **60**), five alcohols (compounds **13**, **80**, **82**, **98** and **99**), and the rest are organic acids and organic acid esters, triterpenoids, coumarins, alkaloid, phenol, amide and glycoside. The contents of above components were similar in these two herbs.

### 3.2. Biomarker Discovery for HD and HC

**Figure 3:**
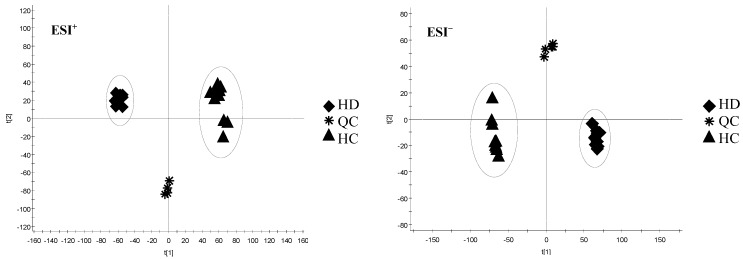
The PCA of HC and HD in positive mode (ESI^+^) and negative mode (ESI^−^). HD: *Hedyotis diffuse* Willd. HC: *Hedyotis corymbosa* (L.) Lam. QC: Quality Control.

PCA, a classic unsupervised lowering-dimension pattern recognition model, can be used to select distinct variables and to find potential biomarkers. It was firstly established based on the spectra of HD and HC samples to discern the presence of inherent similarities in mass spectral profiles as displayed in Figure 3. Two parameters, R^2^ (cum) and Q^2^ (cum), are commonly used to assess the quality of the PCA model, with values close to 1.0 indicative of good fitness and predictive ability. In the present study, R^2^X (cum) and Q^2^ (cum) were 0.6909 and 0.6257, respectively, indicating good fitness and prediction of the constructed PCA model. 

Based on the obtained PCA score plots (Figure 3), the 20 samples were obviously divided into two main groups according to different species (HD and HC). The HD samples were noticeably overlapping, which indicates good similarity among them, and this result was also observed for HC samples. Meanwhile, the HD group and the HC group were completely separated, indicating that these two species herbs could be differentiated. The QC samples were between the two species, which came from the fact that they were mixed volumetrically in 50%. 

In order to distinguish HD from HC, OPLS-DA models were built in both positive and negative modes. OPLS-DA score plot, S-plot, variable trend and VIP (variable importance in the projection) values were obtained to understand which variables are responsible for separation [109].

**Figure 4:**
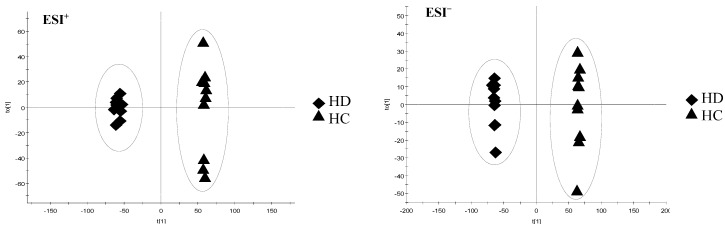
The OPLS-DA of HC and HD in positive mode (ESI^+^) and negative mode (ESI^−^).

As shown in Figure 4, OPLS-DA models were constructed to discriminate the difference under the already established separation between different groups based on the PCA results. Each model has 2 score components (HD and HC). These scores are weighted averages of the original ones, hence providing a good summary. In addition, these scores display the separation of the groups in both ESI^+^ and ESI^−^ modes. The scores t[1] (*x*-axis) and to[1] (*y*-axis) are the two most important new variables in summarizing and separating the data. Each point in the plot corresponds to an observation. The groups are shown in different shapes and the separation of the groups is easily visible in t[1]. The to[1] score values show the variation within each class. This variation can either be caused by biological variation or by systematic changes in the experimental setup.

**Figure 5:**
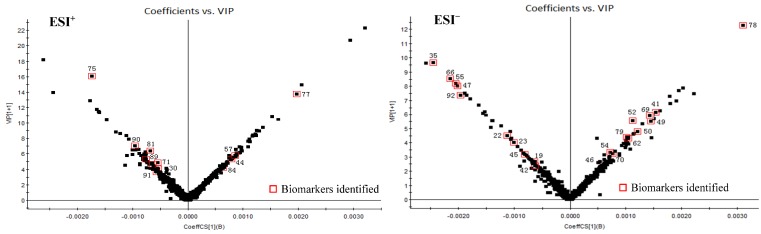
The OPLS-DA/Coefficients vs. VIP of HC and HD in positive (ESI^+^) and negative mode (ESI^−^).

**Figure 6:**
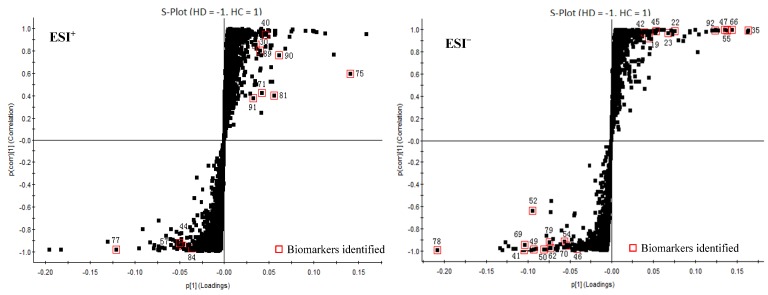
The OPLS-DA/S-Plot of HC and HD in positive mode (ESI^+^) and negative mode (ESI^−^).

**Figure 7:**
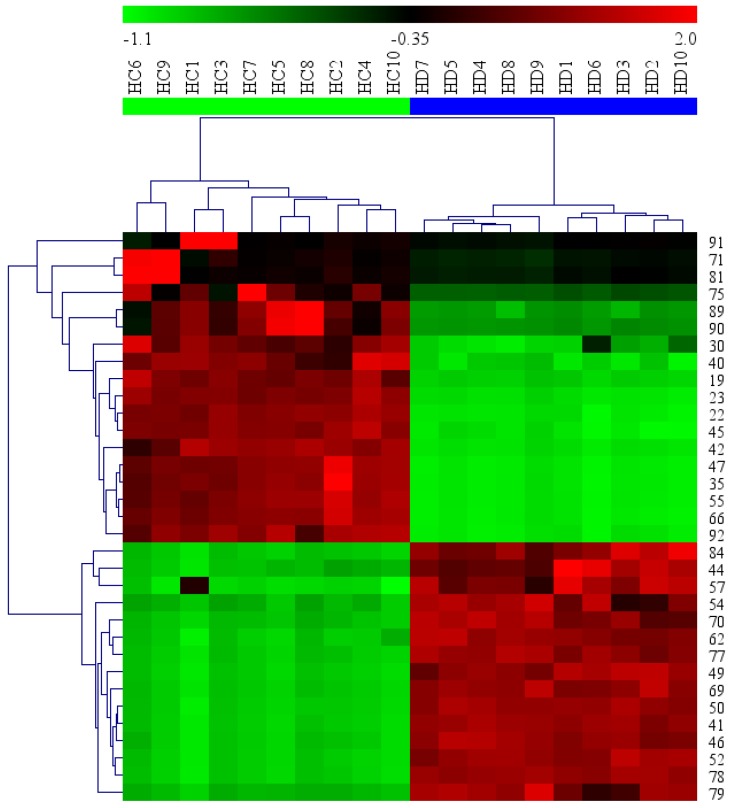
Heatmap visualizing the intensities of potential biomarkers.

Figure 5 displays the variable importance (VIP) versus the PLS-regression coefficients. Important X-variables have large positive VIP values and large positive or negative coefficient values. The covariance p[1] and correlation p(corr)[1] loadings from a two class OPLS-DA model were shown here in S-Plot format (Figure 6). The points are Exact Mass/Retention Time pairs (EMRTs). The upper right quadrant of the S-plot shows those components which are elevated in HC, the control group, while the lower left quadrant shows EMRTs elevated in HD, the treated group. The farther along the *x*-axis the greater the contribution to the variance between the groups, while the farther the *y*-axis the higher the reliability of the analytical result. Based on VIP values (VIP > 4) (Figure 5) and *p* values (*p* < 0.05) [110] from univariate analysis, and the identification of components from HC and HD (Table 2), 33 robust known biomarkers enabling the differentiation between HD and HC were discovered and marked in S-plots (Figure 6). In order to systematically evaluate the biomarkers, a heatmap was generated from these biomarkers (shown in Figure 7), which shows distinct segregation between two species.

## 4. Discussion

There are 109 and 104 compounds characterized from HC and HD respectively. Sixty compounds were identified in ESI^−^ mode and 53 compounds were identified in ESI^+^ mode. According to the BPI chromatograms of HC and HD, it seems that ESI^−^ ionization mode is better than ESI^+^ based on the quantity and the responses of the identified compounds, but it is still necessary to run the ESI^+^ mode because some compounds showed better respond than in ESI^−^ mode. 

It was revealed that HD and HC differed in their chemical composition according to the HPLC analysis [19]. It was also indicated that 6-*O*-(*E*)-*p*-coumaroyl scandoside methyl ester and 6-*O*-(*E*)-*p*-coumaroyl scandoside methyl ester-10-*O*-methyl ether were the main components of HD. In 2007, Liang et al. reported that HD and its substitutes could be identified based on HPLC chemical fingerprints and mass spectrometric analysis [25]. MS combined with UV spectra and literature values was used to obtain the chemical information. As a result, four compounds, asperuloside, 6-*O*-(*E*)-*p*-coumaroyl scandoside methyl ester, 6-*O*-(*E*)-*p*-coumaroyl scandoside methyl ester-10-methyl ester and 6-*O*-*p*-feruloyl scandoside methyl ester were recommended to be used as chemical markers for quality evaluation and chemical authentication of HD and its substitutes. In addition, scandoside methyl ester detected in the chromatograms of HC can be used as the characteristic peaks [25]. Furthermore, a previous report found that hedyotiscone A could be used to differentiate HC from HD using TLC method [22]. In our study, asperuloside, 6-*O*-(*E*)-*p*-coumaroyl scandoside methyl ester-10-methyl ester, scandoside methyl ester, 6-*O*-*p*-feruloyl scandoside methyl ester and hedyotiscone A were shared in HC and HD, but the reported result concerning 6-*O*-(*E*)-*p*-coumaroyl scandoside methyl ester was consistent with our findings.

In the other record, another marker compound, 10(*S*)-hydroxypheophytin a, isolated with a yield of 22 mg from 600 g of HC, was identified exclusively in HD [23]. It is a pity that it was not be detected under our experimental conditions. Similarly, (9*R*,10*S*,7*E*)-6,9,10-trihydroxyoctadec-7-enoic acid, isolated with a yield of 47.9 mg from 20 kg of HC, was reported to be used to differentiate HC from HD [26]. It was not be detected under our experimental conditions either.

In this study, 33 known compounds enabling the robust differentiation between HC and HD were detected. For HC, there were 18 potential biomarkers, including three iridoids (**23**, **55**, **66**), eight flavonoids (**30**, **35**, **40**, **42**, **47**, **71**, **75**, **81**), two tannins (**19**, **45**), two ketones (**22**, **91**), one alcohol (**92**), two monoterpenes (**89**, **90**). Among these potential biomarkers, the contents of nine components (**19**, **22**, **23**, **30**, **35**, **40**, **45**, **66**, **92**) in HC were much greater than in HD. Compounds **42**, **47**, **55**, **71**, **75**, **81**, **89**, **90** and **91** could be detected only in HC. It’s worth mentioning that two iridoids, compounds **55** (hedycoryside B) and **66** (hedycoryside A), with high responses in UPLC-MS might be used for rapid identification of HC. For HD, there were 15 potential biomarkers including two iridoids (**52**, **50**), eight flavonoids (**41**, **44**, **49**, **54**, **57**, **62**, **79**, **84**), one tannin (**46**), one ketone (**70**), and three anthraquinones (**69**, **77**, **78**). Among them, the contents of eleven components (**41**, **44**, **46**, **49**, **52**, **57**, **62**, **70**, **77**, **78**, **79**) in HD were much higher than those in HC. Compounds **50**, **54**, **69** and **84** were detected only in HD. In addition, two anthraquinones, compounds **69** (1,3-dihydroxy-2-methylanthraquinone) and **78** (2-hydroxy-3-methylanthraquinone) with high responses in UPLC-MS might be used for rapid identification of HD.

However, there are still some unresolved issues. Firstly, the pharmaceutical effects associated with these identified compounds should be screened in the future. Secondly, as shown in BPI chromatograms, though 113 compounds were identified, there are still some unidentified components. Further research should be carried out based on the formula of these unknown compounds. Thirdly, source material is not seasonable as it was collected during summer time. Fourthly, collecting HC and HD in the same area may be the better way for comparison. But in this study, Haikou City for HC and Fuzhou City for HD were visited. To some extent, the collection of these samples might be used as negative controls for another species because it could eliminate the influence of the region on the analysis of the sample. But unfortunately, the regional factor should not be considered as there should be more samples per region.

## 5. Conclusions

Under the optimized conditions, a total of 109 chemical compounds with different structural types were identified from HC and 104 from HD. The similarities and differences between these two herbs were also highlighted in the paper. Various structural patterns including iridoids, flavonoids, organic acids and organic acid esters, tannins, alcohols, ketones, coumarins, anthraquinones, monoterpenes, triterpenoids were presenting in these two herbs, of which there were 80 shared compounds in HC and HD. There is quite a difference in the parent structures types between HC and HD. A total of 33 robust biomarkers enabling the differentiation between HC and HD were discovered. For HC and HD, 18 and 15 potential biomarkers, respectively, were identified in this paper. Two iridoids, hedycoryside B (compound **55**) and hedycoryside A (**66**) might be used for rapid identification of HC, and two anthraquinones, 1,3-Dihydroxy-2-methylanthraquinone (compound **69**) and 2-Hydroxy-3-methylanthraquinone (**78**) might be used for rapid identification of HD based on their presence and content. Actually, these solid biomarkers are recommended for further use in the recognition and distinction between HC and HD. The results provided reliable characterization profiles to identify these two herbs and to clarify the fundamental pharmacological substances. Different chemical compositions will inevitably lead to different biological effects of HC and HD in clinical application. HC should not be used as substitute of HD. The results provided data on the chemical constituents of HC and provide a reference for the quality control of HD in the aspect of quantitative determination.

## Figures and Tables

**Table 1:** The list of the tested samples from China.

Sample No.	Source	Collection Time
HC 1	Guangzhou City, Guangdong Province, China; market	15 September 2016
HC 2		26 August 2017
HC 3	Haikou City, Hainan Province, China; market	11 August 2017
HC 4	Nanning City, Guangxi Province, China; field	8 July 2016
HC 5		5 July 2017
HC 6	Kunming City, Yunnan Province, China; market	13 August 2016
HC 7		1 September 2017
HC 8	Shenzhen City, Guangdong Province, China; cultivated	28 September 2017
HC 9	Luoding County, Guangdong Province, China; market	24 August 2016
HC 10		12 July 2017
HD 1	Nanning City, Guangxi Province, China; field	12 July 2016
HD 2		20 July 2016
HD 3	Luoding County, Guangdong Province, China; market	13 August 2016
HD 4		15 July 2017
HD 5	Guangzhou City, Guangdong Province, China; market	13 July 2017
HD 6	Shenzhen City, Guangdong Province, China; cultivated	21 September 2016
HD 7		21 August 2017
HD 8	Kunming City, Yunnan Province, China; market	8 August 2016
HD 9	Fuzhou City, Fujian Province, China; field	22 August 2017
HD 10		13 September 2017

**Table 2:** Compounds identified from HD and HC by UPLC-QTOF-MS^E^.

No.	t_R_ (min)	Formula	Calculated Mass (Da)	Theoretical Mass (Da)	Mass Error (ppm)	MS^E^ Fragmentation	Identification	Source	Ref.
**1**	0.61	C_7_H_12_O_6_	192.0633	192.0634	−0.4	191.0560 [M−H]^−^, 129.0190 [M−C_2_H_2_O_2_]^−^, 127.0407 [M−OH−COOH]^−^	Quinic acid	HC, HD	s
**2**	0.69	C_4_H_6_O_5_	134.0216	134.0215	0.9	133.0144 [M−H]^−^, 115.0037 [M−OH]^−^, 71.0150 [M-OH-COOH]^−^	2-hydroxy-succinic acid	HC, HD	[35]
**3**	0.75	C_6_H_8_O_7_	192.0270	192.0270	0.0	191.0197 [M−H]^−^, 173.0085 [M−OH]^−^, 117.0193 [M−OH−CH_2_COOH]^−^, 111.0089 [M−2 × OH−COOH]^−^, 101.0247 [M−2 × COOH]^−^, 89.0250 [M−CH_2_COOH−COOH]^−^	Citric acid	HC, HD	s
**4**	0.80	C_9_H_10_O_4_	182.0582	182.0579	1.2	227.0564 [M+HCOO]^−^, 165.0558 [M-OH]^−^, 153.0555 [M-CHO]^−^, 137.0611 [M−OH−CHO]^−^, 125.0244 [M−2CH_3_−CHO]^−^	Syringaldehyde	HC, HD	a
**5**	0.93	C_19_H_19_N_3_O	305.1505	305.1528	7	328.1397 [M+Na]^+^, 132.0812 [M−C_10_H_9_N_2_O]^+^, 117.0626 [M−C_11_H_12_N_2_O]^+^, 107.0503 [M−C_11_H_10_N_2_-NCH_3_]^+^	Wuchuyuamide I	HC, HD	[36]
**6**	0.94	C_16_H_20_O_10_	372.1051	372.1057	−1.4	371.0978 [M−H]^−^, 315.0723 [M−C_3_HO]^−^, 167.0712 [M−Glu−COOH]^−^, 153.0192 [M−Glu−C_3_HO]^−^, 123.0451 [M−Glu−C_3_HO_3_]^−^	Deacetyl asperuloside	HC, HD	[37]
**7**	1.11	C_22_H_18_O_10_	442.0866	442.0900	−7.8	443.0938 [M+H]^+^, 319.0772 [M−C_6_H_5_O_3_]^+^, 145.0255 [M−C_13_H_10_O_7_]^+^	(+)-Epicatechol 3-gallate	HC, HD	[38]
**8**	1.20	C_17_H_24_O_11_	404.1318	404.1319	−0.2	449.1300 [M+HCOO]^−^, 353.0872 [M−OH−OCH_3_]^−^, 247.1184 [M−OH−C_6_H_5_O_3_]^−^, 241.0720 [M−Glu]^−^, 211.0610 [M−OCH_3_−Glu]^−^	Scandoside methyl ester	HC, HD	s
**9**	1.34	C_11_H_10_O_5_	222.0519	222.0528	−4.0	223.0592 [M+H]^+^, 209.0418 [M−CH_3_]^+^, 191.0318 [M−OH−CH_3_]^+^, 181.0501 [M−C_2_H_3_O]^+^, 179.0680 [M−COOH]^+^, 163.0364 [M−OH−C_2_H_3_O]^+^	4-*O*-acetyl-caffeic acid	HC, HD	[39]
**10**	1.34	C_10_H_10_O_4_	194.0568	194.0579	−5.8	195.0641 [M+H]^+^, 181.0501 [M−CH_3_]^+^, 179.0680 [M−OH]^+^, 163.0364 [M-CH_3_−OH]^+^, 149.0581 [M−COOH]^+^, 145.0256 [M−OH−OCH_3_]^+^	3-Hydroxy-4-methoxycinnamic acid	HC, HD	[40]
**11**	1.44	C_16_H_22_O_10_	374.1207	374.1213	−0.6	419.1189 [M+HCOO]^−^, 357.1190 [M−OH]^−^, 343.0975 [M−CH_2_OH]^−^, 313.0909 [M−2 × CH_2_OH]^−^, 257.0671 [M−C_5_H_4_O_3_]^−^	Geniposidic acid	HC, HD	s
**12**	1.45	C_8_H_8_O_3_	152.0490	152.0473	9.2	175.0382 [M+Na]^+^, 136.0598 [M−OH]^+^, 119.0494 [M−2 × OH]^+^, 91.0561 [M−OH−COOH]^+^	4-Hydroxybenzeneacetic acid	HC, HD	[41]
**13**	1.46	C_10_H_14_O_5_	214.0840	214.0841	−0.4	213.0768 [M−H]^−^, 195.0657 [M−OH]^−^, 181.0498 [M−CH_2_OH]^−^, 177.0554 [M−2 × OH]^−^, 163.0395 [M−OH−CH_2_OH]^−^, 151.0397 [M−C_2_H_5_O_2_]^−^, 149.0593 [M−3 × OH−CH_3_]^−^	Guaiacyl glycerol	HC, HD	[42]
**14**	1.56	C_18_H_24_O_12_	432.1260	432.1268	−1.8	431.1187 [M−H]^−^, 269.0663 [M−Glu]^−^, 165.0552 [M−Glu−OH−C_3_H_5_O_2_]^−^	Asperulosidic acid	HC, HD	[43]
**15**	1.83	C_16_H_18_O_9_	354.0939	354.0951	−3.4	355.1011 [M+H]^+^, 163.0383 [M−quinic acid]^+^, 145.0264 [M−quinic acid−OH]^+^	Chlorogenic acid	HC, HD	s
**16**	1.90	C_7_H_12_O_6_	192.0632	192.0634	−0.7	191.0632 [M−H]^−^, 173.0445 [M−OH]^−^, 137.0239 [M−2 × OH]^−^, 121.0291 [M−4 × OH]^−^	1,3,4,5-Tetrahydroxycyclohexanecarboxylic acid	HC, HD	a
**17**	2.02	C_33_H_40_O_21_	772.2067	772.2062	0.6	817.2049 [M+HCOO]^−^, 609.1443 [M−Glu]^−^	Kaempferol-3-*O*-sophoroside-7-*O*-*β*-d-glucopyranoside	HC, HD	[44]
**18**	2.09	C_16_H_22_O_11_	390.1152	390.1162	−2.7	389.1079 [M−H]^−^, 209.0454 [M−Glu]^−^, 165.0549 [M−Glu−OH−CH_2_OH]^−^, 121.0658 [M−Glu−OH−CH_2_OH−COOH]^−^	Scandoside	HC, HD	s
**19 #**	2.39	C_20_H_30_O_13_	478.1677	478.1686	−1.8	523.1659 [M+HCOO]^−^, 293.0873 [M−C_9_H_11_O_4_]^−^, 151.0395 [M−furanosyl−Glu]^−^	3,4,5-Trimethoxyphenyl−6-*O*-d-apio-*β*-d-furanosyl-*β*-d-glucopyranoside	HC, HD (HC>>HD)	[45]
**20**	2.50	C_18_H_22_O_11_	414.1157	414.1162	−1.0	459.1139 [M+HCOO]^−^, 367.1029 [M−OH−CH_2_OH]^−^, 251.0555 [M−Glu]^−^, 191.0352 [M−Glu−CH_3_COOH]^−^, 177.0190 [M−Glu−CH_2_COOCH_3_]^−^	Asperuloside	HC, HD	[46]
**21**	2.65	C_9_H_8_O_4_	180.0423	180.0423	0.2	179.0350 [M−H]^−^, 165.0192 [M−CH_3_]^−^, 135.0451 [M−COOH]^−^	(4-Methoxyphenyl)-oxoacetic acid	HC, HD	[47]
**22 #**	3.07	C_15_H_20_O_8_	282.1108	282.1103	1.3	327.1090 [M−H]^−^, 165.0556, 147.0452 [M−Glu]^−^, 121.0294 [M−Glu−CH_3_−CO]^−^	Androsin	HC, HD (HC>>HD)	[48]
**23 #**	3.27	C_17_H_24_O_10_	388.1370	388.1370	0.1	433.1352 [M+HCOO]^−^, 355 [M−OCH_3_]^−^, 353.0876 [M−OH−CH_3_]^−^, 337.0932 [M−OH−OCH_3_]^−^, 225.0770 [M−Glu]^−^, 193.0506 [M−Glu−OCH_3_]^−^	Geniposide	HC, HD (HC>>HD)	s
**24**	3.82	C_14_H_17_NO_6_	295.1052	295.1056	−1.2	340.1034 [M+HCOO]^−^, 167.0346 [M−N−C_6_H_5_−2OH]^−^, 166.0508 [M−3 × OH−C_6_H_5_]^−^	Prunasin	HC, HD	[49]
**25**	4.01	C_15_H_10_O_7_	302.0414	302.0426	−4.3	303.0486 [M+H]^+^, 153.0171 [M−C_8_H_6_O_3_]^+^, 127.0389 [M−C_9_H_6_O_4_]^+^	Moric acid	HC, HD	a
**26**	4.12	C_27_H_30_O_17_	626.1493	626.1483	1.6	625.1420 [M−H]^−^, 609.1424 [M−OH]^−^, 595.1373 [M−CH_2_OH]^−^, 400.0883 [M−OH−CH_2_OH−Glu]^−^, 300.0282 [M−Glu]^−^	Quercetin−3-sophoroside	HC, HD	[50]
**27**	4.14	C_43_H_48_O_25_	964.2508	964.2485	2.4	963.2435 [M−H]^−^, 903.2227 [M−CH_2_OH]^−^, 757.1849 [M−C_11_H_11_O_4_]^−^, 625.1419 [M−C_11_H_11_O_4_−Glu]^−^	Quercetin-3-*O*-(6-*O*-feruloyl-*β*-d-glucopyranosyl)-(1→2)-*β*-d-galactopyranosyl-(1→2)-*β*-d-glucopyranoside	HC, HD	a
**28**	4.15	C_15_H_18_O_8_	326.1002	326.1002	0.0	371.0984 [M+HCOO]^−^, 163.0403 [M−Glu]^−^, 119.0504 [M−Glu−COOH]^−^	trans-*p*-Coumaric acid-4-*O*-glucoside	HC, HD	[51]
**29**	4.19	C_19_H_26_O_12_	446.1421	446.1424	−0.7	491.1403 [M+HCOO]^−^, 371.0986 [M−OCH_3_−C_2_H_3_O]^−^, 283.0824 [M−Glu]^−^, 163.0403 [M−Glu−OCH_3_−C_3_H_5_O_2_]^−^, 119.0504 [M−Glu−OH−C_5_H_8_O_4_]^−^	Daphylloside	HC, HD	[52]
**30 #**	4.24	C_21_H_20_O_11_	448.1000	448.1006	−1.3	449.1072 [M+H]^+^,415.1006 [M−2 × OH]^+^, 397.0920 [M−3 × OH]^+^, 287.0490 [M−Glu]^+^, 137.0587 [M−Glu−C_7_H_3_O_3_]^+^	Luteolin 7-*O*-*β*-d-glucopyranoside	HC, HD (HC>>HD)	s
**31**	4.39	C_26_H_28_O_14_	564.1486	564.1479	1.3	563.1414 [M−H]^−^, 403.1260 [M−OH−C_9_H_6_O_2_]^−^, 275.0578 [M−OH−CH_2_OH−C_6_H_5_O−apiofuranosyl]^−^	Apiin	HC, HD	[53]
**32**	4.48	C_9_H_10_O_3_	166.0631	166.0630	0.7	165.0558 [M−H]^−^, 147.0451 [M-OH]^−^, 119.0501 [M−COOH]^−^, 103.0556 [M−OH−COOH]^−^	Phloretic acid	HC, HD	[54]
**33**	4.59	C_10_H_8_O_4_	192.0413	192.0423	−4.8	193.0486 [M+H]^+^, 178.0247 [M−CH_3_]^+^, 122.0350 [M−C_3_H_2_O_2_]^+^	Scopoletin	HC, HD	[55]
**34**	4.75	C_11_H_16_O_3_	196.1097	196.1099	−0.2	197.117 [M+H]^+^, 179.1057 [M−OH]^+^, 167.0688 [M−2× CH_3_]^+^, 147.0436 [M−2 × CH_3_-OH]^+^	Loliolide	HC, HD	[56]
**35 #**	4.96	C_26_H_28_O_16_	596.1375	596.1377	−0.4	595.1302 [M−H]^−^, 300.0280 [M−Glu−Xyl]^−^	Isoetin-7-*O*-*β*-d-glucopyranosyl-2′-*O*-*β*-d-xyloypyranoside	HC, HD (HC>>HD)	[57]
**36**	4.98	C_15_H_12_O_7_	304.0573	304.0583	−2.8	349.0555 [M+HCOO]^−^, 195.0294 [M−C_6_H_5_O_2_]^−^, 179.0323 [M−OH−C_6_H_5_O_2_]^−^, 151.0036 [M−C_8_H_7_O_3_]^−^	Dihydroquercetin	HC, HD	[58]
**37**	5.04	C_15_H_10_O_7_	302.0424	302.0427	−0.3	303.0496 [M+H]^+^, 287.0541, 127.0395 [M−C_9_H_6_O_4_]^+^	5,7,8,3′,4′-pentamethoxy Flavonoids	HC, HD	[59]
**38**	5.04	C_27_H_30_O_16_	610.1538	610.1534	0.7	611.1611 [M+H]^+^, 465.1016 [M−Rha]^+^, 303.0493 [M−Glu−Rha]^+^	Rutin	HC, HD	s
**39**	5.34	C_21_H_20_O_12_	464.0948	464.0955	−1.4	463.0876 [M−H]^−^, 301.0353 [M−Glu]^−^	Quercetin-3-*O*-glucopyranoside	HC, HD	[60]
**40 #**	5.34	C_22_H_22_O_10_	446.1212	446.1213	−0.2	447.1285 [M+H]^+^, 429.1118 [M−OH]^+^, 175.0383 [M−Glu−C_6_H_3_O]^+^, 163.0388 [M−Glu−C_6_H_5_−OCH_3_]^+^, 131.0489 [M−Glu−C_7_H_3_O_3_]^+^	Acacetin 7-*O*-*β*-d-glucopyranoside	HC, HD (HC>>HD)	[61]
41 *	5.71	C_37_H_38_O_19_	786.2011	786.2007	0.5	831.1993 [M+HCOO]^−^, 565.1556 [M−CH_2_OH−C_10_H_9_O_3_]^−^, 379.0657 [M−Glu−CH_2_OH−C_6_H_5_O−C_7_H_7_O_2_]^−^	Allivictoside F	HC, HD (HD>>HC)	[62]
**42 #**	5.75	C_20_H_18_O_11_	434.0848	434.0849	−0.2	433.0775 [M−H]^−^, 300.0280 [M−Ara]^−^, 163.0401 [M−Ara−C_6_H_4_O_3_]^−^, 147.0450 [M−H−Ara−C_6_H_4_O_3_−OH]^−^	Quercetin-3-*O*-*β*-Arabinopyranose	HC	[63]
**43**	5.79	C_27_H_30_O_15_	594.1588	594.1585	0.6	593.1515 [M−H]^−^, 285.0403 [M−Glu−Rha]^−^	Kaempferol 3-glucoside-7-rhamnoside	HC, HD	[64]
44 *	5.89	C_28_H_32_O_16_	624.1682	624.1690	−1.3	625.1755 [M+H]^+^, 501.1583 [M−C_6_H_4_O_2_]^+^, 479.1155 [M−Rha]^+^, 465.0997 [M−Rha−CH_3_]^+^, 317.0637 [M−Rha−Glu]^+^	Isorhamnetin-3-rutinoside	HC, HD (HD>>HC)	[65]
**45 #**	5.93	C_20_H_12_O_8_	380.0560	380.0532	6.5	425.0542 [M+HCOO]^−^,163.0399 [M−CO−C_11_H_5_O_5_]^−^	Phelligrindins d-9	HC, HD (HC>>HD)	[66]
46 *	6.10	C_26_H_32_O_11_	520.1941	520.1945	−0.6	565.1923 [M+HCOO]^−^, 501.1766 [M−OH]^−^, 489.1748 [M−CH_2_OH]^−^, 339.1233 [M−Glu]^−^	Matairesinol monoglucoside	HC, HD (HD>>HC)	[67]
**47 #**	6.17	C_36_H_36_O_19_	772.1864	772.1851	1.7	771.1791 [M−H]^−^, 565.1548 [M−*p*-Hydroxy-cinnamic acid−CH_2_OH]^−^	Allivictoside G	HC	[62]
**48**	6.38	C_8_H_14_O_2_	187.1049	187.0977	0.4	187.0977 [M−H]^−^, 169.0871 [M−OH]^−^, 125.0973 [M−OH−COOH]^−^, 97.0663 [M−OH−C_3_H_5_O_2_]^−^	Azelaic acid	HC, HD	[68]
49 *	6.52	C_27_H_32_O_15_	596.1749	596.1741	1.3	595.1676 [M−H]^−^, 549.1621 [M−OH−CH_2_OH]^−^, 387.1073 [M−OH−CH_2_OH−Rha]^−^, 369.0977 [M−2 × OH−CH_2_OH−Rha]^−^, 163.0400 [M−C_18_H_24_O_12_]^−^	Neoeriocitrin	HC, HD (HD>>HC)	[69]
50 *	6.82	C_27_H_32_O_14_	580.1808	580.1792	2.5	625.1790 [M+HCOO]^−^, 529.1359 [M−OH−OCH_3_]^−^, 517.1356 [M−OCH_3_−CH_2_OH]^−^, 417.1204 [M−Glu]^−^, 193.0510 [M−C_17_H_23_O_10_]^−^, 147.0449 [M−OCH_3_−Glu−C_10_H_9_O_4_]^−^	6-*O*-*Z*-*p*-feruloyl scandoside methyl ester	HD	[70]
**51**	6.89	C_26_H_30_O_13_	550.1683	550.1686	−0.6	595.1665 [M+HCOO]^−^, 433.14811 [M−OH−C_4_H_4_O_3_]^−^, 403.13121 [M−C_9_H_7_O_2_]^−^, 387.1093 [M−Glu]^−^, 355.0823 [M−Glu−OCH_3_]^−^	10-*O*-*E*-*p*-courmaroyl scandoside methyl ester	HC, HD	[71]
52 *	7.07	C_26_H_30_O_13_	550.1683	550.1686	−0.6	549.1610 [M−H]^−^, 595.1663 [M+HCOO]^−^, 387.1086 [M−Glu]^−^, 370.0789 [M−Glu−CH_3_]^−^, 193.0503 [M−Glu−OCH_3_−C_9_H_7_O_3_]^−^	6-*O*-*p*-coumaroyl scandoside methyl ester	HC, HD (HD>>HC)	[16]
**53**	7.13	C_27_H_32_O_13_	564.1843	564.1843	0	609.1825 [M+HCOO]^−^, 549.1613 [M−CH_3_]^−^, 387.1086 [M−CH_3_−Glu]^−^, 387.1086 [M−CH_3_−C_10_H_9_O_2_]^−^, 370.0789 [M−2 × CH_3_−C_10_H_9_O_2_]^−^, 337.1070 [M−OCH_3_−OH−Glu]^−^	6-*O*-(*E*)-*p*-coumaroyl scandoside methyl ester-10-methyl ester	HC, HD	[72]
54 *	7.18	C_27_H_32_O_14_	580.1800	580.1792	1.3	579.1727 [M−H]^−^, 399.1051 [M−Glu]^−^, 223.0604 [M−Glu−C_10_H_12_O_4_]^−^	Nobiletin-3-*O*-*β*-d-glucoside	HD	[73]
**55 #**	7.58	C_24_H_28_O_12_	508.1581	508.1581	0.1	553.1563 [M+HCOO]^−^, 345.0977 [M−Glu]^−^, 223.0602 [M−Glu−C_7_H_4_O_2_]^+^	Hedycoryside B	HC	[74]
**56**	7.88	C_15_H_10_O_7_	302.0438	302.0426	3.9	303.0511 [M+H]^+^, 287.0549 [M−OH]^+^, 153.0181 [M−C_8_H_6_O_3_]^+^, 152.0565 [M−C_7_H_4_O_4_]^+^	Quercetin	HC, HD	s
57 *	7.91	C_15_H_10_O_6_	286.0489	286.0477	−3.7	287.0540 [M+H]^+^,163.0361 [M−C_6_H_4_O_3_]^+^,149.0589 [M−C_6_H_4_O_3_−OH]^+^, 131.0487 [M−C_6_H_4_O_3_−2 × OH]^+^	Kaempferol	HC, HD (HD>>HC)	s
**58**	7.94	C_23_H_26_O_11_	478.1471	478.1475	−0.9	477.1398 [M−H]^−^, 355.1035 [M−benzoic acid]^−^, 315.0879 [M−Glu]^−^, 285.0406 [M−Glu−C_2_H_3_]^−^, 241.1076 [M−OH−benzoic acid−C_3_H_2_O_3_]^−^	Hedycoryside C	HC, HD	[13]
**59**	8.12	C_24_H_28_O_12_	508.1585	508.1581	0.7	553.1567 [M+HCOO]^−^, 345.0976 [M−Glu]^−^, 207.0655 [M−Glu−benzoic acid]^−^, 137.0245 [M−Glu−benzoic acid−C_4_H_4_O_2_]^−^	10-*O*-benzoyl scandoside methyl ester	HC, HD	[43]
**60**	8.88	C_14_H_12_O_4_	244.0738	244.0736	0.9	245.0811 [M+H]^+^, 227.0693 [M−OH]^+^, 135.0429 [M−C_6_H_3_O_2_]^+^, 119.0493 [M−C_6_H_3_O_2_−OH]^+^, 95.0512 [M−C_8_H_5_O_2_]^+^	Piceatannol	HC, HD	[75]
**61**	9.26	C_17_H_14_O_7_	330.0750	330.0740	3.2	331.0823 [M+H]^+^, 315.0485 [M−CH_3_]^+^, 301.0679 [M−OCH_3_]^+^, 207.0647 [M−OH−C_6_H_5_O_2_]^+^	5,3′,4′-Trihydroxy-6,7-dimethoxy flavonoids	HC, HD	[76]
62 *	9.23	C_28_H_34_O_15_	610.1909	610.1898	1.8	609.1836 [M−H]^−^, 401.1232 [M−OH−OCH_3_−Rha]^−^, 193.0513 [M−2 × Rha−C_6_H_3_O]^−^, 177.0557 [M−2 × Rha−C_6_H_3_O_2_]^−^	Hesperidin	HC, HD (HD>>HC)	s
**63**	9.62	C_20_H_30_O_5_	350.2078	350.2093	−4.2	351.2151 [M+H]^+^, 293.2123 [M−C_2_H_4_O_2_]^+^, 275.1999 [M−C_2_H_4_O_2_−OH]^+^, 257.1917 [M−C_2_H_4_O_2_−OH−OH]^+^, 105.0713 [M−C_6_H_6_O_3_−C_6_H_12_O_2_]^+^	14-Andrographolide	HC, HD	[77]
**64**	9.62	C_16_H_28_O_2_	252.2113	252.2089	8.7	275.2006 [M+Na]^+^, 195.1389 [M−C_4_H_8_]^+^, 155.1050 [M−C_7_H_14_]^+^, 151.1110 [M−C_6_H_12_O]^+^	7-Hexadecenoic acid-16-hydroxy-*O*-lactone	HC, HD	a
**65**	9.98	C_11_H_10_O_3_	190.0625	190.0630	−2.8	191.0697 [M+H]^+^, 177.0533 [M−CH_3_]^+^, 159.0427 [M−CH_3_−OH]^+^, 105.0348 [M−C_6_H_5_O]^+^	6-Methoxy-8-methyl coumarin	HC, HD	s
**66 #**	10.00	C_24_H_28_O_11_	492.1634	492.1632	−0.4	537.1616 [M+HCOO]^−^, 329.1028 [M−Glu]^−^, 207.0622 [M−Glu−C_7_H_5_O]^−^, 195.0664 [M−Glu−OCH_3_−C_7_H_5_O]^−^, 163.0397 [M−Glu−OCH_3_−C_8_H_7_O_2_]^−^	Hedycoryside A	HC, HD (HC>>HD)	[13]
**67**	10.08	C_11_H_16_O_2_	180.1145	180.1150	−2.9	181.1218 [M+H]^+^, 163.1114 [M−O]^+^, 121.1022 [M−C_2_HO_2_]^+^	5,6,7,7*α*-Tetrahydro-4,4,7*α*-trimethyl-2(4*H*)-benzofuranone	HC, HD	[77]
**68**	10.31	C_15_H_10_O_4_	254.0589	254.0579	3.7	255.0661 [M+H]^+^, 240.0411 [M−CH_3_]^+^, 224.0466 [M−OCH_3_]^+^	Alizarin 1-methyl ether	HC, HD	s
69 *	10.64	C_15_H_10_O_4_	254.0579	254.0579	0	253.0506 [M−H]^−^, 224.0477 [M−CH_2_OH]^−^	1,3-Dihydroxy-2-methylanthraquinone	HD	[78]
70 *	11.03	C_15_H_8_O_4_	252.0424	252.0423	0.7	251.0352 [M−H]^−^, 223.0399 [M−O−CH]^−^, 207.0449 [M−COO]^−^	Sanlengdiphenyllactone	HC, HD (HD>>HC)	s
**71 #**	11.65	C_21_H_22_O_8_	402.1311	402.1315	−0.8	403.1384 [M+H]^+^, 387.1084 [M−CH_3_]^+^, 373.0905 [M−2 × CH_3_]^+^, 359.1092 [M−CH_3_−OCH_3_]^+^	Chuan Nectein	HC	[79]
**72**	11.82	C_15_H_16_O_4_	260.1065	260.1049	6.3	261.1138 [M+H]^+^, 205.0499 [M-C_4_H_7_]^+^, 190.0262 [M-C_5_H_9_]^+^, 177.0543 [M-C_5_H_9_O]^+^, 162.0316 [M-OCH_3_-C_5_H_9_]^+^	5-Prenyloxy-7-methoxycoumarin	HC, HD	a
**73**	11.85	C_20_H_26_O_4_	330.1805	330.1831	−7.8	331.1878 [M+H]^+^, 149.0953 [M-OH-C_10_H_13_O_2_]^+^, 131.0489 [M-OH-CH_3_-C_10_H_13_O_2_]^+^, 135.0803 [M-OCH_3_-C_10_H_12_O_2_]^+^, 121.0646 [M-OCH_3_-C_12_H_17_O_2_]^+^	Dihydroguaiac acid	HC, HD	[80]
**74**	11.87	C_15_H_14_O_4_	258.0889	258.0892	−1.3	259.0962 [M+H]^+^, 244.0707 [M-CH_3_]^+^, 229.0480 [M-2 × CH_3_]^+^, 227.0684 [M-OCH_3_]^+^, 217.0474 [M-C_3_H_5_]^+^, 212.0444 [M-CH_3_-OCH_3_]^+^	Hedyotiscone A	HC, HD	[81]
**75 #**	12.11	C_19_H_18_O_6_	342.1103	342.1103	−0.2	343.1175 [M+H]^+^, 327.08434 [M−CH_3_]^+^, 313.06864 [M−2 × CH_3_]^+^, 299.08954 [M−CH_3_−OCH_3_]^+^, 285.07454 [M−2 × CH_3_−OCH_3_]^+^	5,6,7,4′-Tetramethoxyflavone	HC	s
**76**	12.11	C_16_H_28_O_3_	268.2056	268.2038	6.2	291.1949 [M+Na]^+^, 217.1566 [M−CH_3_−OH]^+^, 132.0863 [M−OH−C_2_H_5_−C_4_H_7_O_2_]^+^	13-Hydroxy-9,11-Hexadecandienoic acid	HC, HD	b
77 *	12.41	C_16_H_12_O_4_	286.0731	268.0736	−0.4	269.0804 [M+H]^+^, 254.0557 [M−CH_3_]^+^, 251.06537 [M−OH]^+^, 239.0689 [M−OCH_3_]^+^, 225.0540 [M−OCH_3_−CH_3_]^+^	Methylisotropine-1-methylether	HC, HD (HD>>HC)	a
78 *	12.44	C_15_H_10_O_3_	238.0630	238.0630	0.2	237.0558 [M−H]^−^, 224.0471 [M−CH_3_]^−^, 208.0518 [M−OH−CH_3_]^−^	2-Hydroxy-3-methylanthraquinone	HC, HD (HD>>HC)	[82]
79 *	12.49	C_15_H_10_O_5_	270.0524	270.0528	−1.6	269.0451 [M−H]^−^, 237.0555 [M−2 × OH]^−^	5-Dehydroxykaempferol	HC, HD (HD>>HC)	[83]
**80**	12.51	C_17_H_24_O_3_	276.1730	276.1725	1.8	277.1803 [M+H]^+^, 259.1608 [M−OH]^+^, 231.1774 [M−CH_3_−2 × OH]^+^, 213.1633 [M−CH_3_−3 × OH]^+^, 203.1776 [M−3 × OH−C_2_H_3_]^+^, 201.1612 [M−3 × OH−C_2_H_5_]^+^	(10*E*)1,10-Heptadeca-diene-4,6-diyne-3,8,9-triol	HC, HD	[84]
**81 #**	12.74	C_22_H_24_O_9_	432.1411	432.1420	−2.0	433.1484 [M+H]^+^, 418.1231 [M−CH_3_]^+^, 403.0998 [M−2 × CH_3_]^+^, 388.0763 [M−3 × CH_3_]^+^, 385.0857 [M−CH_3_−OCH_3_]^+^, 372.1131 [M−2 × OCH_3_]^+^, 357.0934 [M−CH_3_−2 × OCH_3_]^+^	3′,4′,5′,5,6,7,8-Seven-methoxyflavone	HC	[85]
**82**	12.80	C_17_H_24_O_2_	260.1774	260.1776	−0.8	305.1756 [M+HCOO]^−^, 135.0813 [M−C_3_H_7_−C_5_H_5_O]^−^, 125.0969 [M−C_2_H_5_−C_7_H_5_O]^−^, 121.0656 [M−C_4_H_9_−C_5_H_5_O]^−^	Fakalinediol	HC, HD	[86]
**83**	13.35	C_30_H_48_O_5_	488.3497	488.3502	−0.9	533.3479 [M+HCOO]^−^, 291.1956 [M−C_12_H_20_O_2_]^−^, 195.1029 [M−C_19_H_29_O_2_]^−^, 171.1025 [M−C_21_H_33_O_2_]^−^	3*β*,19*α*,23-Trihydroxyurs-12-en-28-oic acid	HC, HD	[87]
84 *	13.36	C_17_H_14_O_6_	314.0793	314.0790	0.8	315.0866 [M+H]^+^, 300.0618 [M−CH_3_]^+^, 282.04958 [M−OCH_3_]^+^, 111.04458 [M−CH_3_−C_10_H_6_O_4_]^+^	5,3′-Dihydroxy-7,4′-dimethoxyflavone	HD	[88]
**85**	13.96	C_27_H_28_N_2_O_4_	444.2060	444.2049	2.5	445.2133 [M+H]^+^, 385.1887 [M−C_2_H_3_O_2_]^+^, 224.1062 [M−C_12_H_13_NO_3_]^+^, 194.1172 [M−C_16_H_13_NO_2_]^+^, 134.0970 [M−C_2_H_3_O_2_−C_16_H_13_NO_2_]^+^	Gold Amide Alcohol Ester	HC, HD	[89]
**86**	14.44	C_17_H_32_O_2_	268.2398	268.2402	−1.4	313.2380 [M+HCOO]^−^, 251.2019 [M−CH_3_]^−^, 183.1388 [M−C_6_H_13_]^−^, 129.0918 [M−C_10_H_17_]^−^	Methyl cis-9-hexadecenoate	HC, HD	a
**87**	14.81	C_18_H_34_O_4_	314.2460	314.2457	0.9	337.2352 [M+Na]^+^, 139.1118 [M−C_9_H_18_O_3_]^+^, 125.09614 [M−C_10_H_20_O_3_]^+^	Dibutyl sebacate	HC, HD	a
**88**	14.81	C_18_H_30_O_2_	278.2244	278.2246	−0.8	279.2316 [M+H]^+^, 249.1834 [M−C_2_H_5_]^+^, 217.1935 [M−CH_3_−COO]^+^, 191.1801 [M−C_4_H_6_O_2_]^+^, 163.1483 [M−C_6_H_10_O_2_]^+^	9,12,15-Octadecatrienoic acid	HC, HD	[90]
**89 #**	15.25	C_26_H_32_O_6_	440.2193	440.2199	−1.4	441.2266 [M+H]^+^, 389.2315 [M−C_3_H_2_O]^+^, 340.1657 [M−C_3_H_2_O−C_2_H_3_O]^+^, 147.0437 [M−C_17_H_25_O_4_]^+^	Isofeterin	HC	[91]
**90 #**	15.25	C_20_H_28_O_4_	332.2016	332.1988	7.9	355.1908 [M+Na]^+^, 241.1946 [M−OH−CH_2_OH−COO]^+^, 217.1189 [M−OH−CH_2_OH−CH_3_−C_4_H_8_]^+^, 161.1320 [M−OH−CH_2_OH−CH_3_−C_6_H_5_O_2_]^+^	14-Deoxy-11,12-dihydroandrographolide	HC	[92]
**91 #**	15.69	C_15_H_22_O	218.1659	218.1671	−5.5	219.1731 [M+H]^+^, 163.1106 [M−C_4_H_7_]^+^, 161.0935 [M−CH_3_−C_3_H_6_]^+^	*α*-Turmerone	HC	a
**92 #**	15.87	C_15_H_28_O_2_	240.2090	240.2089		285.2072 [M+HCOO]^−^, 223.2068 [M−OH]^−^	Isodonsesquitin A	HC, HD (HC>>HD)	[93]
**93**	16.02	C_16_H_30_O_2_	254.2251	254.2246	1.8	277.2143 [M+Na]^+^, 137.1316 [M−C_4_H_9_−CH_2_COOH]^+^, 109.1012 [M−C_4_H_9_−C_3_H_6_COOH]^+^	*Z*-11-Hexadecenoic acid	HC, HD	[94]
**94**	16.02	C_20_H_28_O_3_	316.2025	316.2038	−4.1	317.2098 [M+H]^+^, 289.1787 [M−C_2_H_4_]^+^, 277.2151 [M−C_2_H_2_O]^+^, 251.1930 [M−C_4_H_4_O]^+^, 235.1667 [M−C_5_H_7_O]^+^, 221.1503 [M−CH_3_−C_5_H_7_O]^+^	7*β*-Senecioyloxyoplopa-3(14)*Z*,8(10)-dien-2-one	HC, HD	a
**95**	16.03	C_34_H_58_O_4_	530.4316	530.4335	−3.4	553.4208 [M+Na]^+^, 483.3400 [M−3CH_3_]^+^, 317.2060 [M−OCH_3_−C_13_H_27_]^+^, 315.1595 [M−2 × CH_3_−C_13_H_27_]^+^, 313.1703 [M−OH−CH_3_−C_13_H_27_]^+^	Ferulic acid esters lignoceric	HC, HD	a
**96**	16.23	C_16_H_30_O_2_	254.2258	254.2246	4.6	277.2151 [M+Na]^+^, 137.1329 [M−C_2_H_5_−C_4_H_6_O_2_]^+^, 123.1168 [M−C_2_H_5_−C_5_H_8_O_2_]^+^, 111.1171 [M−C_8_H_14_O_2_]^+^	Palmitoleic acid	HC, HD	[95]
**97**	16.23	C_20_H_28_O_3_	316.2021	316.2038	−5.5	317.2094 [M+H]^+^, 301.2068 [M−OH]^+^, 277.2147 [M−C_2_H_2_O]^+^, 259.2029 [M−CH_3_−COOH]^+^, 215.1763 [M−C_2_H_2_O−COOH]^+^, 141.0911 [M−C_11_H_15_]^+^	Terminalic acid	HC, HD	[96]
**98**	16.61	C_20_H_26_O_3_	314.1859	314.1882	−7.2	315.1932 [M+H]^+^, 159.1158 [M−OH−C_8_H_9_O_2_]^+^, 133.1005 [M−C_10_H_13_O_3_]^+^, 147.1165 [M−OH−C_9_H_11_O_2_]^+^	Oxyphyllacinol	HC, HD	[97]
**99**	16.91	C_20_H_26_O_3_	314.1854	314.1882	−8.9	315.1927 [M+H]^+^, 191.1040 [M−OH−C_8_H_9_]^+^, 173.1307 [M−OH−C_7_H_7_O_2_]^+^, 135.0799 [M−OH−OCH_3_−C_10_H_13_]^+^	Neonootkatol	HC, HD	[98]
**100**	17.08	C_17_H_30_O_2_	266.2646	266.2646	0.1	311.2228 [M+HCOO]^−^, 183.1387 [M−C_6_H_12_]^−^, 249.2224 [M−OH]^−^	7,10-Dienylhexadecanoic acid methyl ester	HC, HD	a
**101**	17.37	C_18_H_32_O_3_	296.2355	296.2351	1.3	295.2283 [M−H]^−^, 277.2176 [M−OH]^−^, 233.2262 [M−O−COOH]^−^, 183.1024 [M−CH_3_−5×CH_2_−2×CH]^−^,125.0968 [M−OH−C_10_H_17_O]^−^, 123.1180 [M−O−CH_2_COOH−C_7_H_13_]^−^	Coronaric acid	HC, HD	[99]
**102**	17.37	C_18_H_32_O_3_	296.2355	296.2351	1.3	295.2283 [M−H]^−^, 277.2176 [M−OH]^−^, 233.2262 [M−O−COOH]^−^, 125.0968 [M−C_10_H_17_O_2_]^−^, 123.1180 [M−COOH−C_8_H_15_O]^−^	Vernonia acid	HC, HD	[100]
**103**	17.39	C_30_H_46_O_4_	470.3398	470.3396	0.3	471.347 [M+H]^+^, 455.3448 [M−OH]^+^, 437.3382 [M−2 × OH]^+^, 425.3421 [M−COO]^+^, 420.2712 [M−2 × CH_3_−OH]^+^, 409.3449 [M−OH−COO]^+^, 383.3309 [M−CH_3_−CO−COO]^+^	Caryophylloside	HC, HD	[101]
**104**	17.87	C_20_H_28_O_3_	316.2019	316.2038	−6.2	317.2092 [M+H]^+^, 235.1672 [M−C_5_H_6_O]^+^, 189.1622 [M−C_5_H_6_O−COOH]^+^, 179.1418 [M−OH−CH_3_−C_7_H_8_O]^+^	Saurufuran B	HC, HD	[102]
**105**	17.87	C_18_H_28_O_2_	276.2088	276.2089	−0.3	277.2161 [M+H]^+^, 235.1672 [M−C_3_H_6_]^+^, 217.1967 [M−CH_2_COOH]^+^, 207.1729 [M−OH−C_3_H_6_]^+^, 189.1623 [M−C_3_H_6_COOH]^+^	Stearidonic acid	HC, HD	[103]
**106**	15.99	C_18_H_30_O_3_	294.2197	294.2195	0.7	293.2124 [M−H]^−^, 275.2016 [M−OH]^−^, 211.1340 [M−C_6_H_12_]^−^, 185.1180 [M−C_8_H_14_]^−^, 182.1305 [M−OH−C_7_H_13_]^−^	(*E*,*E*)-9-Oxooctadeca-10,12-dienoic acid	HC, HD	[104]
**107**	18.61	C_20_H_28_O_3_	316.2021	316.2038	−7.0	317.2089 [M+H]^+^, 283.1680 [M−OH−CH_3_]^+^, 259.2034 [M−CH_3_−COOH]^+^, 235.1680 [M−C_5_H_5_O]^+^	Saurufuran A	HC, HD	[103]
**108**	18.61	C_16_H_30_O_2_	254.2270	254.2246	8.7	277.2162 [M+Na]^+^, 179.1405 [M−OH−C_4_H_9_]^+^, 165.1260 [M−OH−C_5_H_11_]^+^, 151.1111 [M−OH−C_6_H_13_]^+^, 125.0963 [M−OH−C_8_H_15_]^+^	Hexadecenoic acid	HC, HD	[105]
**109**	18.68	C_18_H_34_O_3_	298.2511	298.2508	1.1	297.2438 [M−H]^−^, 279.2332 [M−OH]^−^, 155.1076 [M−C_9_H_18_O]^−^	Ricinolic acid	HC, HD	[106]
**110**	19.51	C_20_H_30_O_3_	318.2174	318.2195	−6.6	319.2247 [M+H]^+^, 239.1776 [M−COOH−CH_2_OH]^+^, 233.193 [M−C_4_H_3_O_2_]^+^, 189.1630 [M−OH−C_6_H_7_O_2_]^+^	Andrograpanin	HC, HD	[107]
**111**	21.64	C_30_H_48_O_3_	456.3579	456.3604	−4.8	501.3561 [M+HCOO]^−^, 340.2808 [M−2 × OH−C_6_H_12_]^−^, 277.2159 [M−C_12_H_20_O]^−^, 223.2062 [M−COOH−C_14_H_19_]^−^	Ursolic acid	HC, HD	s
**112**	21.72	C_28_H_48_O_2_	416.3678	416.3654	5.3	439.357 [M+Na]^+^, 342.3004 [M−OH−C_4_H_9_]^+^, 327.2377 [M−2CH_3_−C_4_H_9_]^+^, 277.2119 [M−C_10_H_21_]^+^, 249.1820 [M−CH_3_−C_11_H_23_]^+^	*γ*-Tocopherol	HC, HD	[108]
**113**	23.33	C_19_H_38_O_4_	330.2776	330.2770	1.6	353.2668 [M + Na]^+^, 313.2733 [M−OH]^+^, 283.2593 [M−2 × OH−CH_3_]^+^, 269.2161 [M−OH−C_3_H_7_]^+^, 239.2376 [M−C_3_H_5_O_3_]^+^	Palmitin	HC, HD	a

* Characteritic component in HD; # Characteritic component in HC; s: Identified with reference substance. a: Compared with spectral data obtained from Wiley Subscription Services, Inc. (USA); b: Compared with NIST Chemistry WebBook; HD: *Hedyotis diffuse* Willd.; HC: *Hedyotis corymbosa* (L.) Lam.
